# Mechanisms and Effect of Increased Physical Activity on General and Abdominal Obesity and Associated Metabolic Risk Factors in a Community with Very High Rates of General and Abdominal Obesity

**DOI:** 10.3390/antiox12040826

**Published:** 2023-03-28

**Authors:** Salah Gariballa, Ghada S. M. Al-Bluwi, Javed Yasin

**Affiliations:** Internal Medicine, College of Medicine & Health Sciences, United Arab Emirates University, Al Ain P.O. Box 17666, United Arab Emirates

**Keywords:** antioxidants, oxidative damage, inflammation, obesity, type 2 diabetes, physical activity

## Abstract

Background: The growing prevalence of obesity and related type 2 diabetes is reaching epidemic proportions in the UAE. Physical inactivity is one of the possible factors linking obesity to diabetes and other related complications. However, the molecular mechanisms through which physical inactivity is contributing to increased obesity-related pathologies are not clear. Aims: to measure the effects of increased physical activity on obesity and related metabolic risk factors. Materials and Methods: We investigated the effects of physical activity on body weight, waist circumference (WC) and metabolic risk factors in 965 community free-living Emirati subjects. Physical activity, dietary intake, antioxidant enzymes and markers of oxidative damage and inflammation were measured both at baseline and follow up. A validated questionnaire was used to assess occupation and leisure-related physical activity. We compared metabolic risk factors between subjects stratified by physical activity levels. The Cox proportional hazards analysis was used to determine the independent effects of increased physical activity on presence and absence of obesity, body weight and waist circumference (WC) change at follow up. Results: A total of 965 community free-living subjects [801 (83%) females, mean (SD) age 39 ± 12 years] were recruited and followed up with for a period of 427 ± 223 days. Using WHO cut-of-points for body mass index (BMI), 284 (30%) subjects were overweight and 584 (62%) subjects were obese, compared to 69 (8%) at normal body weight. We found men to be more physically active than women at both leisure and work times. BMI, hip circumference, total body fat, HDL and inflammatory markers (us CRP, TNF) were significantly higher in female subjects, whilst fat free-mass, WC, blood pressure and HbA1c were higher in male subjects (*p* < 0.05). Hypertension and diabetes were more common in male subjects compared to female subjects (*p* < 0.05). Increased physical activity both at baseline and follow up were associated with decreased BMI, WC and inflammatory markers, including us-CRP and TNF. Increased physical activity was associated with significant decrease in abdominal obesity in female subjects and general obesity in both male and females after adjusting for important prognostic indicators [hazard ratio (95% CI): 0.531 (0.399, 0.707); *p* < 0.001; 0.475 (0.341, 0.662); *p* < 0.001 respectively]. Conclusion: Our findings suggest that increased physical activity may decrease the risk of obesity and also mitigate the associated oxidative damage and inflammatory responses.

## 1. Introduction

Obesity represents a major public health problem worldwide and is a major risk factor in the etiology of type 2 diabetes, hypertension and cardiovascular disease (CVD) [1,2,3]. In the Gulf region, however, the prevalence of obesity is increasing rapidly, reaching epidemic proportions in some of the Gulf countries [1,2,3,4]. A recent survey of United Arab Emirates (UAE) citizens revealed that the prevalence of obesity and related cardiovascular diseases is very high and therefore needs urgent public health attention [4]. Although increased body mass index (BMI) is used to define obesity in adults because it correlates with the amount of body fat and associated morbidity, recent work suggests that visceral obesity measured using waist circumference is more closely related to morbidity, especially in the Middle East and south East Asia [5,6,7,8,9]. In a recent cross-sectional study, we reported that both elevated BMI and elevated WC are associated with increased cardio metabolic risk factors in obese Emirati women; however, WC is a stronger predictor than BMI [10]. Visceral fat has especially “bad” metabolic actions because it secretes a number of inflammatory markers; some of them have been implicated in the pathologies associated with obesity [3]. Possible mechanisms that relate obesity and diabetes to increased CVD risk include inflammation and oxidative damage. In obese patients, subclinical inflammation has been found to correlate with markers of oxidative stress in adipose tissue, and this may be the mechanism for obesity-related metabolic syndrome, insulin resistance and diabetes mellitus.

UAE society has been through rapid socioeconomic and social changes with urbanization over the last 40 years. Accompanying changes in diet and lifestyle are therefore leading to a growing epidemic of overweight conditions/obesity, type 2 diabetes and other related CVDs. This includes changes in the physical environment that encourage sedentary behavior and changes in the production and availability of food. For example, physical activity has declined and time spent being sedentary has increased. It has been reported that the majority of Arab adolescents failed to meet the guidelines for daily physical activity—85% of girls and 75% of boys aged 13–15 years engaged in less than 60 min of physical activity per day [11]. Despite the alarming rates of increase in obesity and related type 2 diabetes in the UAE, the molecular mechanisms through which physical inactivity is contributing to both general and abdominal obesity and related morbidities are not clear. In a prospective longitudinal cohort study, we investigated the effects of increased physical activity on obesity and associated metabolic risk factors in community free-living subjects.

## 2. Materials and Methods

A convenient sample of community free-living subjects from Al Ain city, serving a total population of 600,000, were approached and invited to take part in the study. Following informed written consent and their recruitment to the study, all subjects had clinical, dietary and physical activity assessments and anthropometric measurements. A fasting 10 mL of blood was taken for measurements of antioxidants, markers of oxidative damage and inflammation, as well as other related clinical, nutritional and biochemical variables at baseline. Individuals with severe chronic clinical or psychiatric disease, participating in other intervention trials, on dietary supplements or taking anti-obesity medications, as well as those unable to give informed written consent, were excluded. The local research ethical committee approved the study.

### 2.1. Measurements

All participants had a baseline clinical assessment, such as demographic and medical data, history of chronic illnesses, smoking and alcohol and drug intake. Anthropometric data, including body weight, height and body mass index (BMI), were measured using a Tanita body composition analyzer. Results were shown on an easy-to-read display screen and printed on a sheet. Waist circumference (WC) was measured to the nearest 0.1 cm using a flexible plastic tape at the mid-point between the lower ribs and iliac crest. 

### 2.2. Measurement of Physical Activity

A validated questionnaire was used to assess occupation and leisure-related physical activity. Data were obtained on the frequency and duration of daily or weekly physical activity sessions for at least 20 min or more, in which subjects became breathless or sweated. Questions were also asked about the number of hours subjects spent in bed (this included time spent reading, watching television or sleeping [12]. A validated short semi-quantitative food frequency questionnaire designed for self-administration, following a brief verbal discussion, was used to assess subjects’ fruit and vegetable intake. It specified the usual frequency of consumption of food items during the previous 12 months and assessed the average weekly nutrient consumption of each individual. The full version of the questionnaire was developed and validated against a 7-day weighted dietary intake. It was also compared with numerous other diets and used in many other studies [13]. Calorie intake was measured in a subgroup of subjects using locally validated 24 h recall, once at baseline assessment and once in follow up visits.

Blood samples: details of the measurement of metabolic risk factors were published before [10]. Briefly, fasting blood samples were drawn into 2 vacutainer tubes, containing potassium EDTA as an anticoagulant. The samples were thoroughly mixed at room temperature and immediately transferred to the laboratory. Both tubes were centrifuged immediately for 10 min at 4000 rotations/min. Plasma and serum were collected and stored at −80 °C for future determinations of biochemical outcome measures. Antioxidants: commercially available Cayman’s colorimetric assay kits from USA-Kit numbers 706002, 707002 and 703102 were used to measure antioxidant enzymes, including glutathione (GSH), superoxide dismutase (SOD), glutathione peroxidase (GPx) and catalase. Inflammatory markers (TNF)—lipid peroxidation: the concentration of the lipid peroxidation product Thiobarbituric Acid Reactive Substances (TBARS) was measured using an assay kit (no, 10009055) from Cayman Chemical Company, Ann Arbor, MI, USA.Protein oxidation: the content of protein-bound carbonyls used to assess the extent of protein oxidation was determined calorimetrically using a reagent kit (10005020) from Cayman Chemical. Circulating levels of renal and liver functions, lipids and high sensitivity C reactive protein (hsCRP) were measured using an automated analyzer Integra 400 Plus (Roche Diagnostics, Mannheim, Germany).

#### Statistical Power of the Study

The sample size of 965 would allow the detection of a true mean difference of 0.8 mmol/L (given the within group SD 5 mmol/L) in plasma fasting blood glucose between subjects who were very physically active and those who were physically inactive. This sample size would also allow the detection of a true mean difference of 0.7 mg/L in hs-CRP, given the within group SD 5 mg/L, with 80% power and a type 1 error probability of ≤0.05.

### 2.3. Statistics and Analysis

Physical activities during occupation and leisure were stratified by intensity of the daily physical activity. Both one-way and two-way ANOVAs and the nonparametric Kruskal–Wallis H test were used to test within- and between-group differences, and *p* value < 0.05 was considered significant. A Cox proportional hazards model was used to examine the influence of physical activity and fruits and vegetables consumption on the probability of general obesity diagnosis (BMI ≥ 30) in both men and women, as well as the presence of abdominal obesity (waist circumference ≥ 80 cm) in only female subjects at follow up after adjusting for other clinical risk indicators, including age, gender, marital status, level of education and fruits and vegetables consumption. Odds of obesity diagnosis at follow up are presented graphically using the Kaplan–Meier hazard curve.

## 3. Results

A total of 965 subjects [801 (83%) females, mean (SD) age 39 ± 12 years] are included in the final analysis. Table 1 shows the baseline physical activity of the study population. The majority of the study population reported very low levels of physical activity during leisure times, with <20% reported to be physically very active. Overall, men were more physically active than women both at leisure and work times. When subjects were asked how often they were physically active for at least 20 min where they became out of breath and sweated, the majority of patients answered less than once or twice a week. This sedentary lifestyle was accompanied by a high prevalence of overweight conditions and obesity—using WHO cut-of-points for body mass index (BMI), 284 (30%) subjects were overweight and 584 (62%) subjects were obese, compared to 69 (8%) at normal body weight.

Table 2 shows baseline characteristics, demographic, anthropometric, blood pressure and biochemical data of all subjects divided by sex. BMI, hip circumference, total body fat, HDL and inflammatory markers (us CRP, TNF) are significantly higher in female subjects, whilst fat free-mass, WC, blood pressure and HbA1c are higher in male subjects (*p* < 0.05).

Hypertension and diabetes were more common in male subjects compared to female subjects (*p* < 0.05).Increased physical activity both at baseline and follow up were associated with decreased BMI, WC and inflammatory markers, including crp and TNF (Table 3 and Table 4). The benefits of increased physical activity in obese subjects was maintained at a mean follow up period of 427 ± 223 days. There was no statistically significant difference in daily calorie intake between those who were physically very active compared with those who were not physically active both at baseline and follow up (*p* > 0.05).

**Figure 1:**
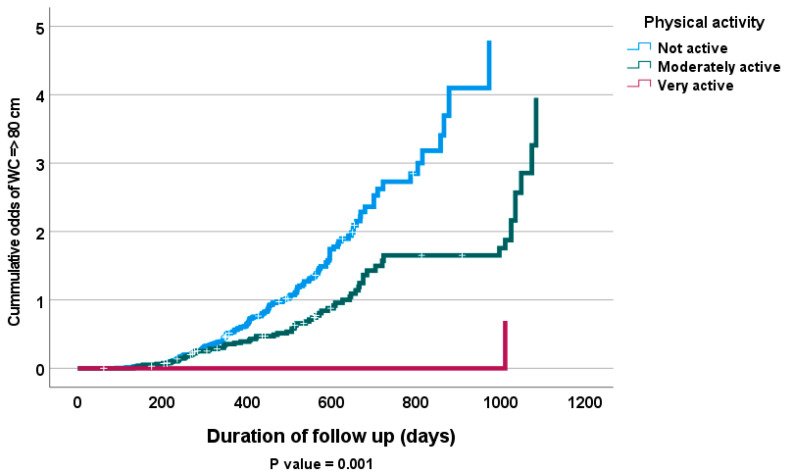
Kaplan–Meier of cumulative odds of WC => 80 cm in female subjects according to levels of physical activity at follow up.

**Figure 2:**
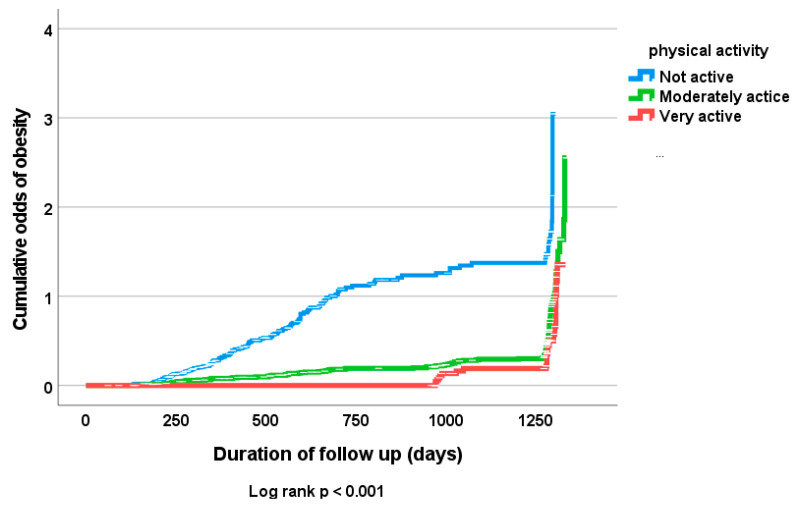
Kaplan–Meier of cumulative odds of diagnosis of obesity (BMI => 30) at follow up in all subjects according to levels of physical activity.

A Cox proportional hazards model was used to examine the influence of physical activity and fruits and vegetables consumption on the probability of general obesity diagnosis (BMI ≥ 30) in both men and women, as well as the presence of abdominal obesity (waist circumference ≥ 80 cm) in only female subjects at follow up after adjusting for other clinical risk indicators, including age, gender, marital status, level of education and fruits and vegetables consumption. Increased physical activity was associated with significant decrease in abdominal obesity in female subjects and general obesity in both male and female subjects after adjusting for important prognostic indicators [hazard ratio (95% CI): 0.531 (0.399, 0.707); *p* < 0.001; 0.475 (0.341, 0.662); *p* < 0.001, respectively] (Table 5 and Table 6). Figure 1 and Figure 2 show significant associations between physical activity and both general obesity in all subjects and abdominal obesity in only females subjects at follow up assessment (*p* = 0.001).

## 4. Discussion

Our results show the majority of the study population reported very low levels of physical activity, with this sedentary lifestyle associated with a high prevalence of overweight conditions and obesity. Men were more physically active compared to women, with BMI, hip circumference, total body fat, HDL and inflammatory markers (us CRP, TNF) being significantly higher in female subjects whilst fat free-mass, WC, blood pressure and HbA1c were higher in male subjects. Hypertension and diabetes were more common in male subjects compared to female subjects (*p* < 0.05).Increased physical activity both at baseline and follow up were associated with decreased BMI, WC and inflammatory markers, including us-CRP and TNF. Increased physical activity was also associated with significant decrease in abdominal obesity in female subjects and general obesity in both males and females after adjusting for important prognostic indicators. 

### 4.1. Physical Activity in the Arab World

A survey of UAE citizens revealed that up to 25% of the population may have diabetes, with the prevalence increasing to 40% amongst those above 55 years of age [4]. Furthermore, associated obesity and hypertension were also found to be common, with diabetes being most common among people with sedentary lifestyles, including professionals and housewives. The report also stated that the growing prevalence of diabetes, physical inactivity and obesity is reaching epidemic proportions in the UAE and needs urgent public health attention [4].Besides obesity, diabetes, hypertension and CVS, there are more than 30 chronic diseases associated with physical inactivity [14]. The WHO 2010 report revealed that chronic non-communicable diseases, such obesity, diabetes, CVS and cancer, are responsible for around 60% of the disability, morbidity and mortality affecting the Arab countries [15].

### 4.2. Physical Activity in the Gulf Countries, Including the UAE

The Gulf countries in particular, including the UAE, have been through rapid socioeconomic and social changes with urbanization over the last 50 years. Accompanying changes in diet and lifestyle are leading to a growing epidemic of overweight conditions/obesity, diabetes and other related cardiovascular diseases. Physical inactivity has been regarded as a major risk factor for obesity and associated pathologies, such as type 2 diabetes, in the Arab world [16]. Overall, lack of physical activity is a key factor in the increasing epidemic of obesity and associated type 2 diabetes in the Middle East region and constitutes a serious public health problem. Most recent survey reports on lifestyle agree that the resident populations of the Gulf region do not get enough exercise to keep themselves healthy. This was confirmed by a systematic review of studies of the current physical activity levels of young residents of the UAE, which revealed that (1) almost a quarter of the young population have a totally sedentary lifestyle with no PA, (2) less than half have been mildly involved in physical activities, (3) around a fifth practice a moderate level of physical activity and (4) a quarter are involved in vigorous physical activity. Additionally, mild physical activity was more common in female adolescents, whereas moderate and high physical activity were significantly higher in male adolescents [17]. Another recent study points to a number of factors and reasons for increased physical inactivity in UAE society, which is a major cause of the obesity epidemic [18]. These factors include the arid desert climate, with high day temperatures for more than half of the year in addition to strong winds and dust storms that often make outdoor walking very uncomfortable. Furthermore, the lack of walking pathways around residential areas, schools and other built environment areas makes it difficult for the public to be engaged in physical activities, including walking. A recent survey of the “Influence of the Built Environment” on physical activity choices among Emirati male and female adolescents found that the built environment was perceived as not being conducive to walking by both female and male adolescents. They had major concerns regarding narrow roads and unmarked crossings, as well as increased traffic density and unsafe driving behavior on roads around schools and neighborhoods [19].

### 4.3. Effect of Physical Activity on Inflammation, Oxidative Damage and Glycemic Control

Despite the increasing prevalence and adverse health effects of physical inactivity, the molecular mechanisms through which physical inactivity negatively impacts human health are not well understood [14]. There is, however, evidence of an association between physical inactivity, insulin resistance and type 2 diabetes, with some estimated 6–10% of all types of type 2 diabetes attributed to physical inactivity [20]. A recent study on clinical and biological factors, including physical activity associated with obesity and type 2 diabetes, has reported increased inflammation with increasing body mass index, fasting blood glucose and decreased adiponectin [21]. A number of inflammatory cytokines secreted by visceral fat have been implicated in obesity-related complications [22]. For example, adiponectin, a hormone secreted by the adipose tissue, has been found to be low in obese patients and plays an important role in the etiology of type 2 diabetes [23]. Besides its insulin sensitizing effects, adiponectin is also known to have anti-inflammatory, antioxidant and cardiovascular modulating effects [24]. Our own research has revealed an inverse association between visceral fat and total adiponectin levels, with visceral fat loss associated with a significant decrease in inflammatory markers and a non-significant increase in total adiponectin levels at follow up [25]. More importantly, some preliminary observations suggest that nutrition interventions, such as calorie restriction, Mediterranean diets and garlic extract administration, may increase adiponectin concentrations [26]. A preliminary feasibility study in 32 normal and overweight subjects randomized between 12-week alternate-day fasting or a control group eating ad libitum revealed reduced total body weight and fat mass coupled with increased plasma adiponectin in the intervention group compared with the control group [27].

Our finding of the inverse association between physical activity and inflammatory markers is important because the ant-inflammatory effects of increased physical activity are thought to be the explanation behind the cardioprotective effect of exercise in subjects with type 2 diabetes and metabolic syndrome [28].

More research work is needed, however, to understand the molecular basis of the relationship between insulin resistance, physical inactivity and sedentary lifestyle. Hitherto, there is substantive evidence supporting the connection between physical inactivity, CVD and all-cause mortality. Physical inactivity is thought to affect endothelial function, microvascular function and macrovascular function, leading to pathological effects on the cardiovascular system [14].

Not only is physical inactivity highly prevalent and associated with chronic illness, so is sedentary behavior, which is defined as long periods of time spent sitting or lying down. It is prevalent and has adverse health effects independent from those of physical inactivity [29]. Furthermore, physical inactivity levels are far more prevalent than currently estimated. Studies revealed that men overestimate their physical activity levels by 44% while women overestimate their physical activity levels by 138% [30].

Another interesting area is the difference in the adverse health impacts of physical inactivity and sedentary lifestyle between men and women, possibly due to hormonal differences between men and menopausal women. A relatively recent study revealed postmenopausal women may obtain less benefit from exercise, but when supplemented with estrogen, that benefit was restored [31]. The relationship between female hormones, physical activity and cardiovascular health is an area for further research.

### 4.4. Other Factors—Diet

Other important risk factors for obesity linked to physical inactivity include diet. For example, diet has been reported by the Global Burden of Disease study to contribute to more diseases such as obesity, diabetes and CVD than physical inactivity, smoking and alcohol combined [32]. Recent studies have convincingly shown that a lifestyle program aimed at reducing body weight, total fat intake and intake of saturates, as well as increasing fiber and physical activity, reduced conversion to diabetes by 58% in a group of obese subjects [33]. Evidence supporting the advantages of a dietary pattern approach rather than focusing exclusively on individual dietary components is also increasing. Typically, such diets have been characterized by higher intakes of fruit, vegetables, legumes, whole grain cereals, poultry and fish. The whole diet approach has been tested in several interventions: the Lyon Diet Heart Study, DASH and the Indian Heart Study [34,35,36,37]. In the Nurses’ Health Study, a diet high in cereal fiber, long chain *n*-3 fatty acids and folate and low in trans fatty acids and glycaemic load (product of glycaemic index and carbohydrate intake) with a high ratio of polyunsaturates to saturates strongly predicted CVD risk. An improvement in these dietary factors explained much of the decline in incidence of CVD in some parts of the world during many years of follow up [38,39].

Recent experimental and human research has pointed to a link between gut microbiomes, weight gain and obesity [40]. Proposed mechanisms through which gut microbiomes affect weight gain and obesity include increased energy production from food, low-grade inflammation and the impact of fatty acid on tissue composition [41]. Several factors impact the diversity and composition of the gut microbiota and are associated with weight gain and obesity, including diet, physical activity, dietary supplementation, medications and bariatric surgery. Research is ongoing on the factors which may improve gut bacterial composition, including the use of probiotics and prebiotics to help treatment and prevention of obesity [40,42].

Overall, recent scientific advances support a number of specific dietary targets to be prioritized for the prevention of obesity-related pathologies, including diabetes and cardiovascular disease. Several are aimed at increasing consumption of healthy foods, with a number reducing the consumption of harmful ones. As an example, the phytochemicals flavonoids, α-linoleic acid and omega-3 fatty acids found in abundance in nuts, fruit, vegetables, olive oil and oily fish rapidly exert positive health effects by attenuating thrombosis and inflammation, besides having strong antioxidant and free-radical scavenging properties.

New knowledge is also coming through about the interactions that occur between diet and life style factors, such as physical activity. For example, whilst low fat diets effectively reduce LDL-cholesterol, they can simultaneously raise triglyceride levels. However, this latter effect can be offset by moderate physical activity or the inclusion of *n*-3 long chain fatty acids in the diet, demonstrating the need for a holistic approach [38].

### 4.5. What Needs to Be Done

With the growing epidemic of general and visceral obesity and related complications, including diabetes and CVD disease, in our society and other similar nations, there is an urgent need for a simple and practical intervention to help reduce this burden. Physical activity, for example, has been shown to reduce the risk of diabetes, hypertension and both cardiovascular and all-cause mortality. The current international recommendation is that adults should accumulate at least 150 min of moderate-intensity physical activity or 70 min of vigorous-intensity physical activity weekly [39]. The alarming rates of physical inactivity among young UAE residents should stimulate policymakers and college/university administrations to plan and promote PA in order to reduce future levels of chronic diseases, such as obesity, type 2 diabetes, hypertension and consequently CVD diseases.

Patients should be encouraged to reduce their inactivity rather than “do more exercise”, which for some people may have negative connotations of team sports and “going to the gym”. Weight loss and long-term weight maintenance will be improved if activity levels can be increased. Step counters may be useful to set daily targets, but their value is unclear. As well as its effect on weight loss, increased physical activity has additional benefits for cardiovascular risk factors, insulin resistance and depression, as well as limiting the loss of lean tissue and contributing to bone health.

Helping someone to change their behavior to prevent or reduce obesity requires a flexible approach tailored to that individual, with encouragement when setbacks inevitably occur [39]. Adopting the core actions proposed by the World Health Organization to counteract obesity could make a good start. These measures include (1) reducing commercial pressure on people (particularly children) to consume high-energy products, (2) reducing fat, sugar and salt in manufactured products, (3) enabling easier and cheaper access to healthy food, (4) introducing measures to improve food and increase physical activity in schools and the workplace, (5) promoting cycling and walking via better urban design and transport policies, (6) creating opportunities in local environments for people to be more physically active in their leisure time and (7) encouraging health services to provide advice on diet and physical activity and promote exclusive breast feeding.

The above actions should involve commitment from the food industry and advertisers and other relevant agencies from the private sector. Other measures, such as street sections that include a wide sidewalk and bike lanes, with minimum lane width and lower speed limits, need to be considered for inclusion. Furthermore, alternative approaches, such as pedestrian bridges for students to circumvent major roads with high speed to reduce exposure to high traffic, should be incorporated in the design manual. In existing neighborhoods, retrofitting and improving alleys’ physical conditions and appearances could also enhance their use and thus support walkability [43].

### 4.6. Limitations and Strengths of the Study

The main limitation of our study is the use of a validated interview questionnaire to evaluate physical activity both at baseline and at follow up as opposed to using records from a physical activity log, a pedometer or training metrics. This may have introduced bias given the reported evidence that both men and women overestimate their physical activity levels by a significant amount [30]. Another limitation is the non-random selection of the study population. Our sample size is large, coming from a society with the second highest prevalence of obesity-related diabetes mellitus in the world. We have adjusted for important lifestyle and prognostic factors in the analysis. Body composition measurements were performed digitally and printed on a sheet with little room for observer error. Biochemical analyses of inflammatory, oxidative and antioxidants were carried out by a laboratory technician not involved in the recruitment or data collection.

## 5. Conclusions

The majority of the study population reported very low levels of physical activity, with a sedentary lifestyle associated with a high prevalence of overweight conditions and obesity. Increased physical activity both at baseline and follow up were independently associated with decreased BMI, WC and inflammatory markers. Public health actions are urgently needed to increase physical activity and reduce sedentary behavior. Coupled with the availability of healthy food choices, this could have enormous public health implications by mitigating obesity-related adverse health effects in our community and worldwide.

## Figures and Tables

**Table 1:** Baseline exercise diary of men and women, number (%).

* Variable		Men (*n* = 164)	Women (*n* = 803)	*p*-Value
How physically active is your leisure time	not very active	33 (20)	239 (30)	<0.023
	moderately active	97 (59)	430 (54)	
	very active	32 (20)	132 (16)	
How physically active is your occupation	not very active	21 (13)	134 (17)	<0.001
	moderately active	61 (37)	244 (30)	
	very active	73 (45)	215 (27)	
	not working	8 (5)	207 (26)	
How many hours per week do you spend doing housework	Mean (SD)	0.6 (1.1)	5.5 (8.5)	<0.001
How often are you physically active for at least 20 min, where you become out of breath and sweat	<1/week	62 (38)	324 (41)	0.697
	1–2/week	33 (20)	128 (16)	
	3–4/week	25 (15)	146 (18)	
	5–6/week	20 (12)	112 (14)	
	7–8/week	17 (10)	78 (10)	
	>8/week	4 (2)	4 (0.5)	
How many hours per day do you usually spend in bed (this includes times spent reading, watching television, sleeping)	<1 h	0	3 (0.5)	<0.019
	1–2 h	2 (1)	11 (1.5)	
	3–4 h	6 (4)	29 (3.5)	
	5–6 h	42 (26)	170 (21)	
	7–8 h	71 (43)	282 (35)	
	>8 h	0	308 (38)	

* Sometimes numbers do not add up because of missing values.

**Table 2:** Baseline clinical, anthropometric, blood pressure and biochemical data of all subjects stratified by sex, mean (SD), unless stated otherwise.

		Men (*n* = 164)	Women (*n* = 801)	*p* Value
Age (years)		44 (14)	38 (11)	0.001
Previous diabetes, *n* (%)		42 (26)	96 (12)	0.001
Previous hypertension, *n* (%)		36 (22)	105 (13)	0.003
Dyslipidemia, *n* (%)		26 (16)	71 (9)	0.352
Smoking	Current	43 (26)	30 (4)	
	Ex-smoker	6 (4)	13 (2)	
	Never	91 (55)	419 (52)	0.001
Body mass index		31.0 (5)	32.4 (6)	0.010
Waist circumference (cm)		103 (15)	99 (13)	0.028
Hip circumference		60 (57)	86 (50)	0.001
Total body fat		30 (9)	38.7 (9)	0.001
Fat free mass		60 (13)	47 (6)	0.001
Systolic BP (mmHg)		130 (18)	120 (13)	0.001
Diastolic BP (mmHg)		78 (9)	74 (10)	0.001
Total cholesterol (mmol/L)		4.86 (1)	4.9 (0.9)	0.626
LDL (mmol/L)		3.3 (0.9)	3.1 (0.9)	0.557
Triglycerides(mmol/L)		1.8 (1.6)	1.3 (0.7)	0.001
HDL (mmol/L)		1.02 (0.3)	1.21 (0.4)	0.001
HbA1c (%)		6.4 (1.6)	5.7 (0.9)	0.001
Glucose (mmol/L)		7.7 (4.6)	8.7 (11)	0.077
Hs C-reactive proteins (mg/L)		2.93 (4.3)	5.13 (5.7)	0.001
TNFα (pg/mL)		5.8 (4.5)	11.0 (5.6)	0.001
Glutathione (GSH) (nM/mL)		4.7 (3)	5.4 (3.7)	0.107
Superoxide dismutase (U/mL)		2.86 (1.4)	3.35 (1.9)	0.038
Catalase (nmol/min/mL)		72 (30)	49 (33)	0.001
Glutathione peroxidase (ng/mL)		189 (84)	122 (98)	0.001
TBARS (nmol/mL)		76 (201)	35 (36)	0.037
Protein carbonyl (nmol/mL)		117 (84)	49 (79)	0.007

**Table 3:** Baseline anthropometric, BP, lipids, antioxidants and markers of oxidative damage and inflammation according to physical activity levels at leisure times, mean (SD), unless stated otherwise.

	Not Active	Moderately Active	Very Active	* *p* Value
Age (years)	38 (12)	41 (12)	41 (11)	0.002
Fruits and vegetables consumption (serving/day)	3.5 (1.6)	4.6 (1.6)	5.2 (1.4)	0.001
BMI	33.0 (6.5)	31.9 (5.9)	31.4 (5.8)	0.011
Waist circumference (cm)	100.6 (13.5)	99.1 (14.1)	99.3 (12.4)	0.445
Systolic BP (mmHg)	120.4 (13.3)	121.9 (15.6)	124.6 (14.9)	0.046
Diastolic BP (mmHg)	74.6 (9.2)	74.8 (10.0)	75.8 (10.5)	0.555
Total cholesterol (mmol/L)	4.7 (0.8)	5.0 (1.0)	5.0 (1.0)	0.021
LDL (mmol/L)	3.0 (0.8)	3.2 (0.9)	3.3 (0.9)	0.004
Triglycerides(mmol/L)	1.2 (0.9)	1.4 (0.9)	1.6 (1.3)	0.016
HDL (mmol/L)	1.1 (0.3)	1.2 (0.4)	1.2 (0.4)	0.000
HbA1c (%)	5.9 (1.2)	5.9 (1.2)	5.9 (1.1)	0.915
Glucose (mmol/L)	8.9 (12.8)	8.5 (9.1)	7.6 (6.7)	0.520
hs-C-reactive proteins (mg/L)	5.1 (5.7)	4.8 (5.7)	3.6 (4.2)	0.082
TNFα (pg/mL)	12.9 (4.1)	9.4 (6.0)	6.4 (5.4)	0.000
TBARS (nmol/mL)	36 (34)	52 (132)	30 (33)	0.117
Protein carbonyl (nmol/mL)	92 (59)	115 (68)	146 (66)	0.001
Glutathione (GSH) (nM/mL)	5.95 (4)	5.18 (3.4)	3.80 (2)	0.002
Glutathione peroxidase (ng/mL)	75 (69)	146 (86)	203 (112)	0.001
Superoxide dismutase (U/mL)	3.71 (2.5)	3.13 (1.7)	2.92 (1.1)	0.044
Catalase (nmol/min/mL)	35 (23)	57 (33)	71 (35)	0.001

* *p* value for differences in anthropometric and metabolic markers between levels of physical activity using one-way ANOVA.

**Table 4:** Follow up anthropometric, BP, lipids, antioxidants and markers of oxidative damage and inflammation according to physical activity levels at leisure times, mean (SD%), unless stated otherwise.

	Not Active	Moderately Active	Very Active	* *p* Value
Fruits and vegetables consumption (serving/day)	3.5 (1.2)	4.3 (1.4)	4.8 (1.1)	0.001
BMI	32.1 (6.0)	29.9 (5.4)	29.0 (4.0)	0.000
Waist circumference (cm)	96.4 (14.6)	93.7 (14.1)	88.0 (0.0)	0.253
Systolic BP (mmHg)	119.4 (10.5)	118.9 (10.8)	113.5 (14.3)	0.388
Diastolic BP (mmHg)	73.8 (8.5)	74.0 (8.3)	84.5 (25.1)	0.004
Total cholesterol (mmol/L)	5.0 (0.9)	5.0 (0.9)	5.1 (0.7)	0.852
LDL (mmol/L)	3.1 (0.8)	3.2 (0.9)	3.4 (0.7)	0.520
Triglycerides (mmol/L)	1.1 (0.6)	1.5 (0.9)	1.3 (0.7)	0.004
HDL (mmol/L)	1.2 (0.3)	1.2 (0.3)	1.2 (0.3)	0.336
HbA1c (%)	5.9 (1.3)	5.7 (1.1)	5.8 (1.1)	0.230
Glucose (mmol/L)	6.4 (6.0)	6.5 (4.0)	6.1 (2.5)	0.928
hs-C-reactive proteins (mg/L)	4.6 (5.4)	4.1 (4.7)	3.0 (3.3)	0.061
TNFα(pg/mL)	7.3 (3.6)	5.9 (3.9)	3.9 (4.0)	0.000
TBARS (nmol/ml	39.9 (85.4)	49.5 (130.4)	59.7 (161.8)	0.588
Protein carbonyl (nmol/mL)	85 (73)	126 (72)	163 (71)	0.001
Glutathione (GSH) (nM/mL)	6.2 (3.8)	6.4 (3.2)	5.8 (5.0)	0.719
Glutathione peroxidase (ng/mL)	79 (84)	123 (50)	138 (39)	0.001
Superoxide dismutase (U/mL)	4.7 (3.3)	3.3 (2.1)	2.8 (0.8)	0.000
Catalase (nmol/min/mL)	53 (73)	71 (30)	80 (24)	0.001

* *p* value for differences in anthropometric and metabolic markers between levels of physical activity using one-way ANOVA.

**Table 5:** The Cox proportional hazards analysis of the relationship between physical activity and other prognostic variables and diagnosis of abdominal obesity (WC => 80 cm) in female subjects at follow up.

	B	SE	Sig.	Exp(B)	95.0% CI for Exp(B)	
					Lower	Upper
Age (years)	0.009	0.008	0.274	1.009	0.993	1.026
Marital status (married, unmarried, divorced)	0.019	0.099	0.847	1.019	0.840	1.237
Level of education (primary, secondary, graduate)	0.012	0.059	0.837	1.012	0.902	1.135
Occupation (employed, unemployed)	−0.018	0.058	0.754	0.982	0.876	1.101
Follow up physical activity (not active, moderately active, very active)	−0.633	0.146	<0.001	0.531	0.399	0.707
Follow up fruits and vegetables consumption (servings/day)	0.022	0.142	0.875	1.023	0.775	1.349

**Table 6:** The Cox proportional hazard analysis of the relationship between physical activity and other prognostic variables and diagnosis of obesity (BMI => 30) at follow up.

	B	SE	Sig.	Exp(B)	95.0% CI for Exp(B)	
					Lower	Upper
Age (years)	0.010	0.009	0.257	1.010	0.992	1.029
Sex (male/female)	0.657	0.299	0.028	1.928	1.073	3.464
Marital status (married, unmarried, divorced)	−0.035	0.112	0.753	0.965	0.776	1.202
Level of education (primary, secondary, graduate)	−0.007	0.067	0.921	0.993	0.872	1.132
Occupation (employed, unemployed)	0.003	0.066	0.967	1.003	0.880	1.142
Follow up physical activity (not active, moderately active, very active)	−0.744	0.169	<0.001	0.475	0.341	0.662
Follow up fruits and vegetables consumption (servings/day)	0.010	0.157	0.949	1.010	0.742	1.374

## Data Availability

Data is contained within the article.
